# The 2009 pandemic (H1N1) viruses isolated from pigs show enhanced pathogenicity in mice

**DOI:** 10.1186/1297-9716-44-41

**Published:** 2013-06-11

**Authors:** Yongtao Li, Wei Zou, Guangmin Jia, Jianjiang Ke, Jiping Zhu, Xian Lin, Hongbo Zhou, Meilin Jin

**Affiliations:** 1 Unit of Animal Infectious Diseases, State Key Laboratory of Agricultural Microbiology, Huazhong Agricultural University, 1 Shizishan Street, Wuhan, Hubei 430070, P.R. China; 2 College of Veterinary Medicine, Huazhong Agricultural University, 1 Shizishan Street, Wuhan, Hubei 430070, P.R. China

## Abstract

Since the emergence of the 2009 pandemic (H1N1) virus (2009/H1N1) in April 2009, cases of transmission from humans to pigs have been reported frequently. In our previous studies, four 2009/H1N1 variants were isolated from pigs. To better understand the phenotypic differences of the pig isolates compared with the human isolate, in this study mice were inoculated intranasally with different 2009/H1N1 viruses, and monitored for morbidity, mortality, and viral replication, cytokine production and pathological changes in the lungs. The results show that all isolates show effective replication in lungs, but varying in their ability to cause morbidity. In particular, the strains of A/swine/Nanchang/3/2010 (H1N1) and A/swine/Nanchang/F9/2010 (H1N1) show the greatest virulence with a persisting replication in lungs and high lethality for mice, compared with the human isolate A/Liaoning /14/2009 (H1N1), which shows low virulence in mice. Furthermore, the lethal strains could induce more severe lung pathological changes and higher production of cytokines than that of other strains at an early stage. Amino acid sequence analysis illustrates prominent differences in viral surface glycoproteins and polymerase subunits between pig isolates and human strains that might correlate with their phenotypic differences. These studies demonstrate that the 2009/H1N1 pig isolates exhibit heterogeneous infectivity and pathogencity in mice, and some strains possess an enhanced pathogenicity compared with the human isolate.

## Introduction

The 2009 pandemic H1N1 influenza virus (designed 2009/H1N1) emerged in April 2009, rapidly spreading in human populations, and developing into the first pandemic virus of the 21^st^ century [1]. Though the world is at the post-pandemic period, 2009/H1N1 virus might pose a potential threat to humans or animals. Epidemiological or serological surveillances identifying the 2009/H1N1 virus in pigs show that it is still on the rise. The first case of 2009/H1N1 infection in pigs was reported in a commercial swine herd in Alberta, Canada, and the pigs subjected to the 2009/H1N1 pig isolates recovered relatively quickly compared with those infected with 2009/H1N1 isolates from humans [2]. Subsequently, the natural infection of pigs with 2009/H1N1 has been reported in more than 10 countries, including China [3,4], Thailand [5], South Korea [6], United Kingdom and others [7,8]. When compared with the sequences of the representative A/California/04/2009 strain (CA/04), the protein sequences of these pig isolates display different amino acid mutations, though they share high homology with those of CA/04 [4,9,10]. More importantly, what should be taken into account is that novel viruses have been generated by the reassortment of 2009/H1N1 with other influenza virus strains circulating in pig populations [11-13], which further confirms the potential threat of 2009/H1N1 to public health.

To date, numerous studies have addressed the pathogenesis of 2009/H1N1 in animal models, including mice [14-16], ferrets [16,17], guinea pigs [18], monkeys [15] and others [19,20]. For instance, the representative CA/04 strain replicates efficiently in nonhuman primates and replicates without clinical symptoms in specific-pathogen free miniature pigs [15]. In the mice model, studies on the infectivity of different 2009/H1N1 isolates show high virus titers on 3 days post infection (dpi) and a slight decrease on 6 dpi in lung tissues although the decrease varies among strains [15,16]. Many investigators have concentrated on the pathogenicity of 2009/H1N1 isolates in a mice model, and found that most of the tested 2009/H1N1 viruses show low lethality in mice, only at the highest dose of 10^6.5^EID_50_ or PFU, though they could cause more severe pathological lesions in lungs than currently seasonal A(H1N1) viruses [14,15,21,22]. Most strikingly, researchers recently isolated some 2009/H1N1 variants with certain amino acid mutations, and infection experiments showed that these variants could induce approximately 40%-100% lethal response in mice even at the lower doses [10,23,24], which indicates that 2009/H1N1 viruses possess a potential phenotypic variability in the evolutionary process.

In our previous studies, four novel 2009/H1N1 viruses were isolated from pigs and primary experiments showed the strains could cause systemic infection in mice, and two strains could induce predominant lethal response in mice, indicating the possibly enhanced pathogenicity of these isolates [4]. Nevertheless, the characterization of these 2009/H1N1 pig isolates in a mice model remains largely unknown. To better understand the comparative pathogenesis of 2009/H1N1 pig isolates compared with the human isolate, in the present study, systematic experiments in a mice model were performed to evaluate the infectivity and pathogenicity of these novel strains, and to further investigate the pathological changes in the lungs and cytokine responses induced by these isolates in comparison with a 2009/H1N1 human isolate. The findings demonstrate that 2009/H1N1 pig isolates exhibit heterogeneous infectivity and pathogencity in mice, and some strains possess an enhanced pathogenicity compared with the human isolate.

## Materials and methods

### Viruses and cells

The influenza A viruses used in this study are shown in Table 1. The four novel viruses were isolated from pigs in our clinical surveillances and conserved at −80°C. A/swine/Nanchang/3/2010 (H1N1) (3/10), A/swine/Nanchang/5/2010 (H1N1) (5/10), and A/swine/Nanchang/6/2010 (H1N1) (6/10) were isolated from tracheal mucus of pigs at the age of about 50 days. A/swine/Nanchang/F9/2010 (H1N1) (F9/10) was isolated from the lung tissue of pigs that showed mild or asymptomatic respiratory signs. Genome sequencing of the four viruses showed that they exhibited more than 99% homology to sequences of CA/04 [4]. A/Liaoning/14/2009 (H1N1) (LN/09) was isolated from the nasal swab of a patient with influenza-like symptoms. All viruses were propagated in Madin-Darby canine kidney (MDCK) cells grown in DMEM supplemented with 10% fetal bovine serum, 1% penicillin-streptomycin and TPCK trypsin (1 μg/mL) (Gibco, Karlsruhe, Germany). The second passage of each virus was used in the following mice experiments. The viral titer was determined by a 50% tissue culture infectious dose (TCID_50_) assay in MDCK cells according to the standard methods [25].

**Table 1:** 2009/H1N1 Influenza viruses used in this study

**Influenza virus strains**	**Name in study**	**GenBank ID**	**Source**	**TCID**_**50**_	**MLD**_**50**_^**a**^
A/swine/Nanchang/3/2010 (H1N1)	3/10	JF275917-JF275924	Tracheal mucus	10^-5.5^	10^3^TCID_50_
A/swine/Nanchang/5/2010 (H1N1)	5/10	JF275933-JF275940	Tracheal mucus	10^-3.2^	NC^b^
A/swine/Nanchang/6/2010 (H1N1)	6/10	JF275941-JF275948	Tracheal mucus	10^-4.4^	NC
A/swine/Nanchang/F9/2010 (H1N1)	F9/10	JF275925-JF275932	Lung tissue	10^-4.48^	10^2.98^TCID_50_
A/Liaoning /14/2009(H1N1)	LN/09	KC683492-KC683499	Nasal swab	10^-5.5^	NC

^a^The 50% mouse lethal dose (MLD_50_) was determined by intranasally inoculating groups of ten mice with serial 10-fold dilutions of each virus in a final volume of 50 μL and calculated by the method of Reed and Muench [25].

^b^NC indicates the MLD_50_ of the strains could not be counted due to low mortality or no lethality for mice.

### Mice infection studies

All experiments with mice were performed according to protocols approved by Biological Studies Animal Care and Use Committee in Hubei province, China (approval number: SCXK 2008–0004).

Six-week-old specific-pathogen-free female BALB/c mice were obtained from the Institute of Laboratory Animal Sciences, Wuhan, China. The mice were lightly anesthetized in a chamber with isoflurane and inoculated intranasally with the virus. The 50% mouse lethal dose (MLD_50_) was determined by intranasally inoculating groups of ten mice with serial 10-fold dilutions of each virus in a final volume of 50 μL. In a separate experiment, 12 mice were infected with 10^3^TCID_50_ (50 μL) of each virus. Body weight and survival percents were monitored daily for 14 days and mice with body weight loss of more than 25% of initial body weight were humanely euthanized with quick cervical dislocation. Six mice in each group were euthanized respectively on 3, 5, 7 and 9 dpi to obtain lung tissues for subsequent quantification of cytokines and pathological investigations. Lungs collected for pathology were inflated with 10% neutral-buffered formalin. In order to determine viral titers in the lungs of infected mice in a dose dependent manner, mice were infected with varying concentrations (10^2^ to 10^5^ TCID_50_) of each virus and sacrificed on 3 and 6 dpi respectively. All animal experiments with 2009/H1N1 viruses were conducted in the biosafety level 2+ containment facility approved by the Chinese Ministry of Agriculture.

### Tissue virus load

In order to detect virus replication in lung tissues of mice infected with 10^3^TCID_50_ (50 μL) of each virus, the lungs of mice were homogenized in 1 mL cold PBS and the exact virus titers were determined using clarified tissue homogenates in 10-day eggs from initial dilutions of 1:10, following serial titration. Fifty percent egg infectious dose (EID_50_) titer for egg-grown stocks was calculated by the method of Reed and Muench [25]. The mean viral titers were determined on 3 and 6 dpi, respectively. To determine viral titers in the lungs of infected mice in a dose dependent manner, total RNA of the lungs was extracted with Trizol reagent (Invitrogen, Carlsbad, CA, USA) according to the manufacturer’s instructions, and then treated with RQ1 RNase-Free DNase (Promega, Madison, USA) to remove potential genomic DNA contamination. Reverse transcription and quantitative real time PCR was performed using universal U12 primer for viral RNA (vRNA) and NP primers (Forward: CAGGAAACGCTGAGATTGAA and Reverse: TGGGTTTTCATTTGGTCTCA) designed in the conserved region of the NP gene, respectively [26]. Absolute quantitative values were calculated using a standard curve which was generated by ten-fold serial dilutions of standard plasmid harboring NP gene of 2009/H1N1 virus. PCR were done in triplicate to guarantee the reproducibility of amplification of the cDNA sample.

### Histopathological analysis

Grossly evident pulmonary changes were visually estimated based upon the percent of virus-affected lesions in each lung lobe as described previously [27]. For histopathological analysis, mice lungs were removed immediately following euthanasia, inflated and fixed with 10% neutral buffered formalin overnight at 4°C. Subsequently, the formalin-preserved lung samples were embedded in paraffin and sectioned. Serial 4-mm sections were stained with Hematoxylin and Eosin (H&E), and examined for pathological changes corresponding to infection. Images were obtained on an Olympus BX-50 light microscope at 10- or 50-fold original magnification.

### Cytokine assays

On 3, 5, 7 and 9 dpi respectively, the lung homogenates from mice inoculated with each virus were prepared to measure the production of IL-6, IL-10, IL-12 (p40), IL-1β, IFN-γ and TNFα by enzyme-linked immunosorbent assay (ELISA) according to the manufacturer’s protocol (Dakewe, Shenzhen, China). Each test had three replicates.

### Sequence analysis

DNA sequences were combined and edited using the Lasergene sequencing analysis software (DNASTAR, Madison, WI, USA). Multiple sequence alignments were performed by DNAMAN software (Version 5.2.2. Lynnon BioSoft, USA). A/California/04/2009(H1N1) was chosen as the referent strain (Table 2).

**Table 2:** The amino acid mutations in the proteins of the 2009/H1N1 pig isolates

**Strains**	**PB2**	**PA**		**HA**^**c**^				**NP**			**NA**		**NS1**
	**588**	**70**	**547**	**103**	**145**	**193**	**239**	**34**	**100**	**344**	**106**	**248**	**123**
CA/04^**a**^	T	A	D	D	S	L	D	G	V	S	V	N	I
LN/09	T	A	D	D	S	L	D	G	V	S	V	N	I
3/10	*I*^**b**^	*V*	*E*	*E*	*P*	L	D	G	*I*	S	*I*	*D*	*V*
5/10	*I*	*V*	*E*	*E*	*P*	L	D	**S**	*I*	S	*I*	*D*	*V*
6/10	*I*	*V*	*E*	*E*	*P*	**I**^**d**^	D	G	*I*	S	*I*	*D*	*V*
F9/10	*I*	*V*	*E*	*E*	*P*	**I**	**G**	G	*I*	**L**	*I*	*D*	*V*

^a^The complete genomic sequences for the referent strain A/California/04/2009 are available in the GenBank database under accession numbers FJ966079.1-FJ966086.1. The genomic sequences of strains used in this study are in the GenBank database and the accession numbers are shown in Table 1.

^b^Italics show the common mutations of 2009/H1N1 pig isolates compared with the human strains.

^c^The HA protein sequence is shown with the 17 amino acids of signaling peptide sequences at the N-terminal.

^d^Bold letters indicate the mutations between the 2009/H1N1 pig isolates.

### Statistical analysis

All experiments were reproducible and carried out in triplicate or quadruplicate. Each set of experiments was repeated at least three times. Statistical analyses were done by one-way ANOVA with Bonferroni multiple comparison test to compare each group of virus with the others using GraphPad Prism version 5 (GraphPad Software Inc., La Jolla, CA, USA). *P* values of < 0.05 were considered to indicate a statistically significant difference between different groups.

## Results

### Characterization of 2009/H1N1 viruses in mice

**Figure 1:**
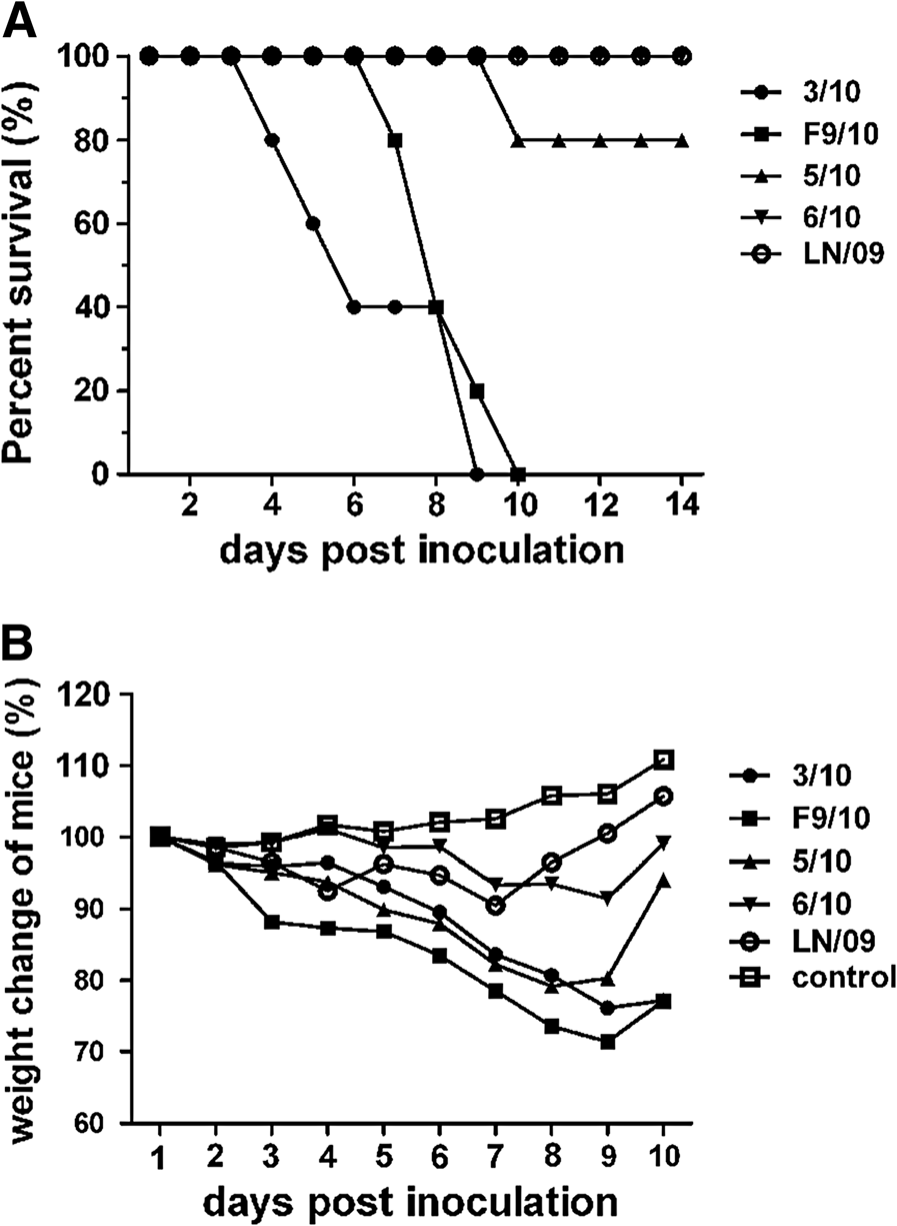
**Weight loss and mortality of mice inoculated with different 2009/H1N1 virues in mice.** Six-week-old BALB/c mice were inoculated intranasally with 10^3^TCID_50_ viruses, with 10 mice per group. The mice that lost 25% of its preinoculation body weight were humanely euthanized with quick cervical dislocation and the data were expressed as the survival percentage of mice infected with 10^3^TCID_50_ viruses (**A**). Body weight of mice infected with of each virus was measured for 14 dpi (**B**).

**Figure 2:**
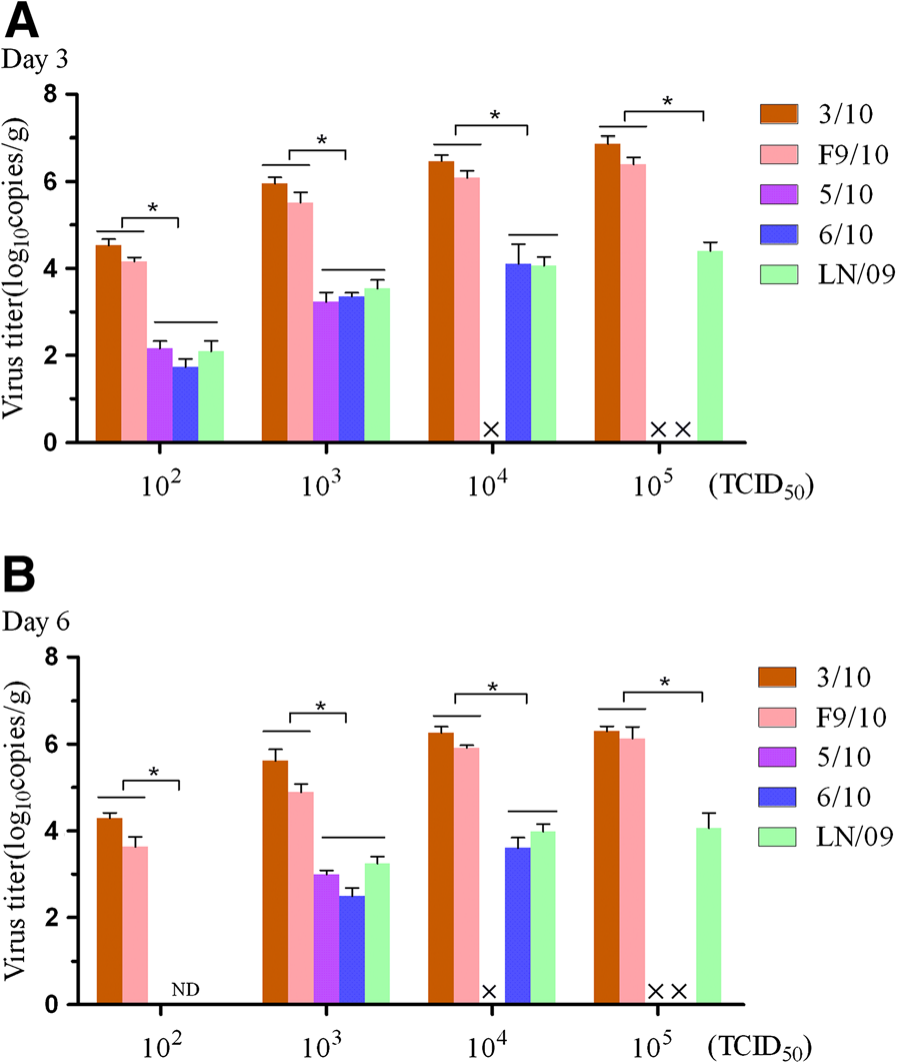
**Comparison of the viral replication in mice of different 2009/H1N1 strains in a dose dependent manner.** Five mice of each group were infected with a dose series of the viruses (10^2^-10^5^ TCID_50_), euthanized at 3 and 6 dpi and the titers in lung homogenates of mice were determined using absolute quantitative PCR on 3 (**A**) and 6 dpi (**B**) respectively. The values were calculated through a standard curve which was generated by ten-fold serial dilutions of standard plasmid harboring NP gene of 2009/H1N1 virus. The results are presented as mean ± SEM of virus copy numbers. × indicates the infection dose in the groups exceeds the content of the original virus. ND means the virus could be not detected. Significant differences were observed at 3/10 and F9/10 compared with 5/10, 6/10 and LN/09 (* *p* < 0.05).

As mentioned above, extensive studies have indicated that most 2009/H1N1 isolated at the early stage had low virulence for mice except infection at higher viral doses (LD_50_ >10^6^EID_50_ or PFU). To understand the lethal characteristics in mice of the 2009/H1N1 pig isolates, in this study, we determined the MLD_50_ for each of the viruses with 10 mice in each group and found that only 3/10 and F9/10 could induce mortality in mice with similar MLD_50_ (10^3^TCID_50_). The human isolate LN/09 did not cause a lethal infection in mice at varying concentrations, which displayed only transient weight reduction until 7 dpi (Table 1). Therefore, we determined the body weight loss and survival percents of mice, and virus titers in the lungs of mice at 10^3^TCID_50_ of each virus. The results show that in contrast to the LN/09, the pig isolates, particularly, 3/10 and F9/10 demonstrate relatively effective infection in mice without prior adaptation and all the mice infected with 3/10 and F9/10 died within 10 dpi (Figure 1A). The virulence of F9/10 was the most prominent and challenge with F9/10 led to nearly 30% body weight loss of initial body weight on 9 dpi. Similarly, mice inoculated with 3/10 suffered more than 20% loss of initial body weight on 9 dpi, whereas the body weight loss of mice infected with 5/10, 6/10 and LN/09 was less than 20% all the time (Figure 1B). To investigate the replication differences of each virus in mice, we determined the virus titers in the lungs from three inoculated mice at 10^3^TCID_50_ in each group. The results show that mice inoculated with the 3/10 and F9/10 viruses possessed significantly higher titers than those of other viruses. The 5/10, 6/10 and LN/09 viruses were detected in the lungs with comparable titers on 3 and 6 dpi (Data not shown). To further determine lung virus titers in a dose dependent manner, we titrated lung homogenates of mice infected with dose series of the viruses (10^2^-10^5^ TCID_50_) by absolute quantitative PCR on 3 and 6 dpi respectively. After data analysis, it was found that the virus titers on 3 dpi were higher than those on 6 dpi, although the decrease of titers varied among strains on 6 dpi. Similarly, the viral titers of 3/10 and F9/10 in lungs were still significantly higher than those of other viruses, regardless of the infection doses and times (Figure 2). The above observations confirmed that 3/10 and F9/10 possess a higher replication than the other viruses. To sum up, both 3/10 and F9/10 could be highly lethal to mice, indicating a characteristic of higher infectivity and virulence than that of the human LN/09 isolate.

### Histological pathology observed in virus infected mice

**Figure 3:**
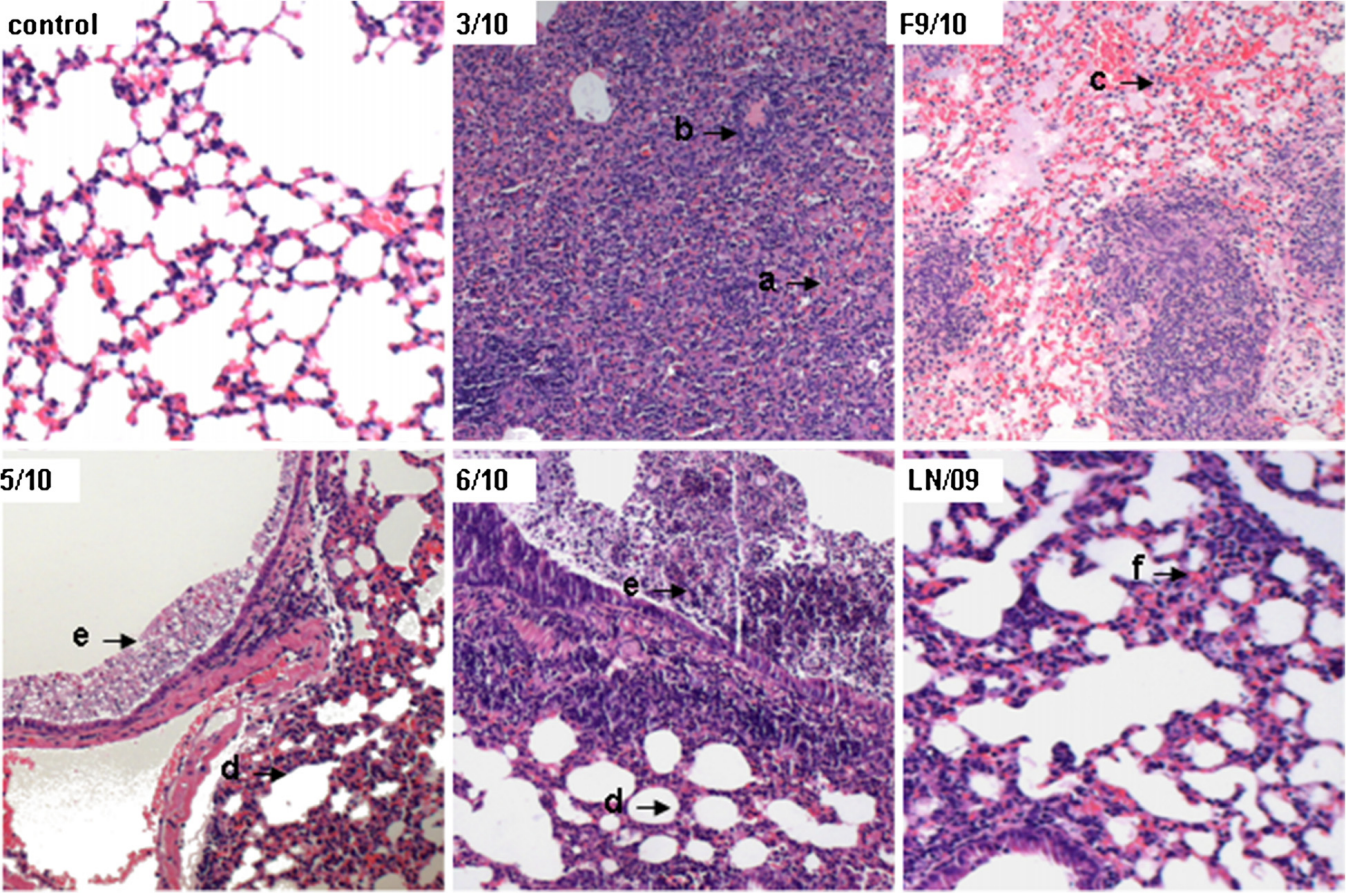
**Comparison of Lung pathology in mice infected with different H1N1/2009 viruses on 5 dpi.** In 3/10-infected mice lungs, signs of acute interstitial pneumonia with infiltration of large amount of lymphocytes (arrow a) and atelectasis (arrow b) were present. Lung tissues in lesion areas of F9/10-infected mice show acute pneumonia with alveolar wall thickening, large amounts of infiltration of inflammatory cells and bleeding (arrow c). The pathological changes of lung tissues infected with 5/10 and 6/10 viruses just show certain alveolar wall thickening (arrow d) and bronchial exudates (arrow e). LN/09-infected mice only show slight alveolar wall thickening in lung lesions (arrow f). Images were obtained on an Olympus BX-50 light microscope at 50-fold original magnifications (50×).

The macroscopic lesions estimated visually suggest that F9/10 infected-mice lungs exhibited the most robust pathophysiology, including dominant pneumonia with increased lung elastance and severe edema formation, with lesions occurring in 100% lung tissue sections on 3 and 5 dpi. In addition, mice inoculated with the 3/10 virus exhibited pathological changes in more than 75% tissue sections on 5 dpi. Moreover, mice infected with 5/10 and 6/10 were associated with lesion occurrence in 20%-40% of lung on 5 dpi. However, mice inoculated with LN/09 virus displayed the mildest pathology (data not shown). In order to examine the microscopic pathological changes in the lungs of mice challenged with different viruses, lung tissues were isolated for HE staining on 3, 5 and 7 dpi respectively. The results show that most of the lungs of mice infected with pig isolates exhibited characteristic pathology of influenza infection, including inflammatory hyperaemia, hemorrhage, edema, and exudative pathological changes. An additional figure file shows the lung pathological changes in more detail (see Additional file 1). On 5 dpi, 3/10-infected mice lungs show acute interstitial pneumonia with infiltration of large amounts of lymphocytes (arrow a) and atelectasis (arrow b), in accordance with the dyspnea and high mortality of infected mice. Similarly, F9/10-infected mice lungs show acute pneumonia with alveolar wall thickening, large amounts of infiltration of inflammatory cells and bleeding (arrow c). Relatively, 5/10 and 6/10 induced pathological changes of the lungs just show certain alveolar wall thickening (arrow d) and bronchial exudate (arrow e). However, LN/09-infected mice show only slight alveolar wall thickening in lung lesions (arrow f) (Figure 3). Collectively, 2009/H1N1 pig isolates could cause severe macroscopic lesions and microscopic pathological changes in the lungs of mice compared to the human isolate.

### Cytokine production following infection with 2009/H1N1 viruses in mice

**Figure 4:**
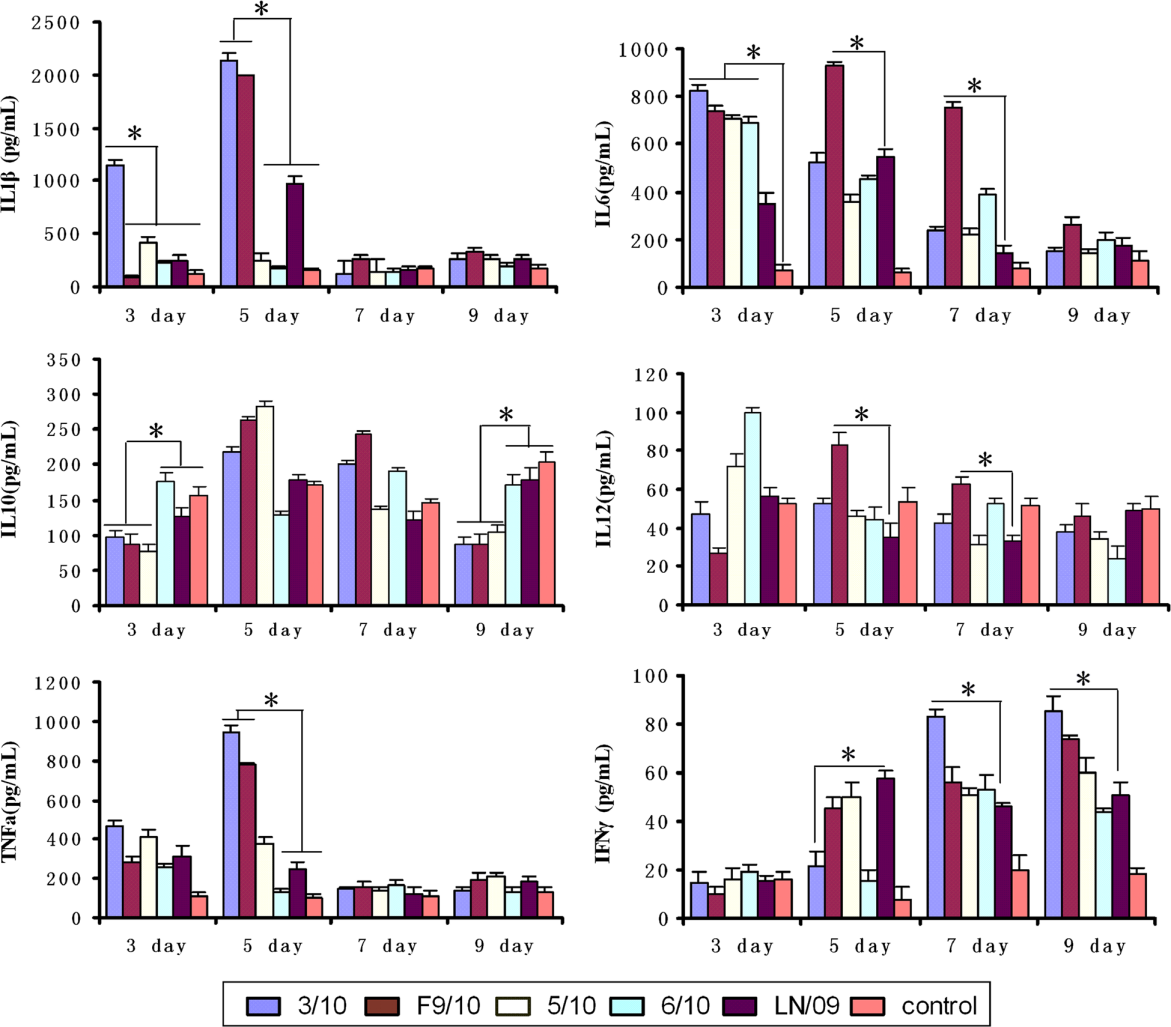
**Cytokine production of lungs in BALB/c mice infected with different H1N1/2009 viruses.** On 3, 5, 7 and 9 dpi respectively, the lung homogenates from mice inoculated with the four 2009/H1N1 pig isolates (3/10, F9/10, 5/10 and 6/10), a human isolate designed LN/09 and control mice were prepared and tested for the production of cytokines IL-1β, IL-6, IL-10, IL-12 (p40), TNFα and IFN-γ by ELISA assay. The results are presented as mean ± SD (*n* = 3) of protein levels.The statistical analysis at each time point was performed compared to data for LN/09 virus or other viruses with lower cytokine levels (* *p* < 0.05).

To determine the inflammatory cytokine production induced by 2009/H1N1 viruses, lungs from mice infected with 10^3^TCID_50_ of the viruses were collected on 3, 5, 7 and 9 dpi, and homogenates were subsequently assayed by ELISA for IL-1β, IL6, IL10, IL12, TNFa and IFN-γ. Among the assayed cytokines, IL-1β, IL6 and TNFa in the lungs of virus-infected mice were up-regulated greater than constitutive levels on 3 and 5 dpi. However, on 7 and 9 dpi, IL-1β, TNFa, IL10 and IL12 in infected mice were not significantly different from those in control mice, except that IL6 and IFN-γ were still higher in infected mice. Although the extent of cytokine induction varied among the strains, there remained some specific characteristics of virus induced cytokine profile. Firstly, in 3/10-infected mice, the production of IL-1β was significantly higher than that of other viruses on 3 and 5 dpi. Secondly, on 5 dpi, IL6 and TNFa were highly increased in F9/10-infected mice compared with other virus-infected mice. In addition, the level of IFN-γ induction was higher in mice infected with 3/10 on 7 and 9 dpi. On 5 dpi, the levels of most cytokines except IFN-γ were higher in F9/10-infected mice than other virus-infected mice, and IL6 was the highest in F9/10-infected mice on 7 dpi. In addition, the 5/10 virus only slightly stimulated the production of IL10 on 5 dpi without predominant changes of other cytokines. In the lungs of 6/10- and LN/09-infected mice, only IL10 induction was relatively higher than that of other viruses. By comparison, the pig isolates, 3/10 and F9/10 could induce higher productions of proinflammatory cytokine TNFa, IL6 and IL-1β than that of the human isolate LN/09 (Figure 4). In summary, most of the proinflammatory cytokines in mice infected with the virulent isolates with lethal characteristics for mice were elevated sharply, and maintained for a longer time.

### Amino acid sequence analysis

Although there is the absence of known virulence markers in 2009/H1N1 virus, such as lysine (K) residue at 627 in PB2 and the multi-basic cleavage site in hemagglutinin (HA), as well as truncated PB1-F2 and NS1 proteins [28], sequence analysis reveals that several common amino acid mutations were found in six protein segments of the four pig isolates based on the sequences of human isolates. In particular, most amino acid differences were presented at the surface glycoproteins of hemagglutinin (HA) and neuraminidase (NA), e.g., S145P mutations in HA and V146I in NA, and polymerase subunits, e.g., A70V and D547E mutations in the PA protein and T588I in PB2 (italics in Table 2). Sequence comparison among the four pig isolates revealed that F9/10 differed from other viruses at three positions, L193I, D239G in HA (also called 176 and 222 sites by deleting signaling peptide sequences) and S344L in NP. The strains of 5/10 and 6/10 had only one mutation at G34S in NP and L193I in HA respectively (bold letters in Table 2). Animal experiments have already shown that 3/10 and F9/10 are more virulent than 5/10 and 6/10 due to efficient viral replication. Possibly, these amino acid residues in NP and HA protein have an important role in the virulence of these novel 2009/H1N1 viruses.

## Discussion

Many studies have explored the prevalence of 2009/H1N1 viruses isolated from pig populations worldwide. The strains from different cases display a significant diversity of pathogenesis in pigs [2,9,19]. Recently, researchers compared the pathogenesis of two 2009/H1N1 viruses, one derived from humans and another from pigs, with a classical swine influenza virus in a pig model. Their studies confirmed that pigs are susceptible to either the swine or human 2009/H1N1 isolates with clear symptoms and an early increase of proinflammatory gene expression [9]. Mice have shown promising potential for elucidating the basic viral pathogenesis of influenza virus. Nevertheless, the comparative research on the pathogenicity and host response of 2009/H1N1 human and pig isolates in the mice model remains largely unknown. To explore this, in this study, mice were inoculated intranasally with the viruses, monitored for body weight loss, morbidity and mortality, and measured for viral replication and cytokine response differences in the lungs. Here, we report that the 2009/H1N1 pig isolates possessed heterogeneous pathogenicity in mice, and the strains of 3/10 and F9/10 could cause high mortality for mice and induce high cytokines in mice lungs compared with the human isolate. However, 5/10 and 6/10 only induced milder manifestations and lower cytokine production in mice with lower mortality than 3/10 and F9/10. Furthermore, mice inoculated with the human isolate LN/09 exhibited the mildest disease and no casualty of mice was found. The histological pathology in the lungs of 3/10- and F9/10-infected mice, which was consistent with the disease outcomes, exhibited the most severe pathological changes, compared to those induced by other viruses.

Generally, disease or death due to influenza virus infection depends on host and viral factors [29]. In terms of host factor, overproduction of cytokines such as TNFa, IL6 and IL8 can result in severe inflammation, such as excessive recruitment of neutrophils and mononuclear cells at the sites of infection, which has been considered to be the basis for the clinical and pathological manifestations of the disease [30]. Previous studies have indicated that during severe high pathogenic avian influenza virus infections, increased expression of proinflammatory mediators and reduced expression of anti-inflammatory mediators in the lungs of hosts correlates closely with severe disease and lethal outcome [31]. For instance, IL-6, a cytokine involved in the induction of fever and the acute phase response has been reported to be correlated with the relative severity of clinical signs in ferrets infected with seasonal H1N1 or H3N2 viruses [32]. In the studies presented here, we used different 2009/H1N1 strains to study the time-course host cytokine responses in a controlled experimental setting, and demonstrate strain-specific or common differences in the induction of inflammatory responses of these viruses. The strains with lethal characteristics, 3/10 and F9/10 could induce higher productions of TNFa and IL-1β than other viruses. Moreover, F9/10 possessed the ability of inducing high expression of IL6 on the long term of infection (Figure 4). In addition, many studies have confirmed that influenza virus uses multiple mechanisms to attenuate the host anti-viral response, allowing for successful infection [33]. In the present study, IFN-γ, as a significant cytokine involved in host defense, was induced at a low level by 3/10 on 3 and 5 dpi and increased only at the late stage of infection, which was consistent with severely pathological changes of the corresponding mice. Likewise, the production of the anti-inflammatory cytokine IL10 was relatively low at the early time of infection in the lethal strain-infected mice compared with that in other viruses infected mice [34]. These results indicated that severely pathological changes in 3/10 and F9/10 infected mice may be partly attributed to the higher expression of pro-inflammatory cytokine and lower production of anti-viral and anti-inflammatory cytokine, though more cytokines need to be investigated in an additional study. In conclusion, the cytokine profiles of mice in response to different viruses may contribute to the different clinical progression and disease outcomes.

In terms of the viral factor, in order to identify a possible correlation between sequence differences and virus pathogenicity in mice, we searched for amino acid mutation between these four pig isolates and human isolates. The pig isolates possessed several common amino acid mutations as shown in Table 2. Although the difference of amino acids did not match the known functional sites associated with pathogenicity in mice, these novel viruses possessed six common amino acid changes in HA, PA, PB2 and NS1 proteins compared with human 2009/H1N1 strains. Some of the above mutations were similar to the molecular changes of 2009/H1N1 virus reported by other researchers [23,35]. It is reported that the HA, PB2, PA and NS genes of 2009/H1N1 are in the classical swine lineage derived from the 1918 Spanish pandemic virus, and many viral mutations associated with the pathogenicity and cross-species transmission of 2009/H1N1 are present in these viral proteins, e.g., D222G in HA [36], T271A in PB2 [37], T552S in PA [38] and E125D in NS1 [39]. Interestingly, in our study, F9/10 had a D222G substitution in HA protein which was absent in other 2009/H1N1 pig isolates submitted to GenBank except the strain of A/swine/Hong Kong/189/2010. Whether the above substitutions in the pig isolates play significant roles in pathogenesis or cross-species transmission, and what these mutations represent in pigs need to be uncovered by ongoing studies. Therefore, the occurrence of possible virulence markers warrant the need for continued systematic surveillance of novel influenza viruses.

In conclusion, since pigs play a significant role in the evolution of influenza viruses and the generation of more virulent variants, more attention should be paid to human-to-animal transmission, even possibly animal-to-human transmission of 2009/H1N1 virus, especially in events involving pig herds.

## Abbreviations

2009/H1N1: 2009 H1N1 influenza virus; CA/04: A/California/04/2009; TCID50: 50% Tissue culture infectious dose; MLD50: Fifty percent mouse lethal dose; dpi: Day post inoculation; EID50: 50% Egg infectious dose; H&E: Hematoxylin and Eosin; ELISA: Enzyme-linked immunosorbent assay.

## Competing interests

The authors declare that they have no competing interests.

## Authors’ contributions

YL, WZ, and MJ designed research, YL, WZ, GJ, JK, JZ performed research, HZ and XL analyzed data, and YL, HZ and MJ wrote the paper. All authors read and approve the final manuscript.

## Supplementary Material

Additional file 1 **Lung pathology in BALB/c mice infected with different H1N1/2009 viruses.** The formalin-preserved lung samples of mice infected with the four H1N1/2009 pig isolates (3/10, F9/10, 5/10 and 6/10), a human isolate designed LN/09 and control mice were embedded in paraffin and sectioned on 3, 5 and 7 dpi. Serial 4-mm sections were stained with Hematoxylin and Eosin (H&E), and examined for pathological changes that corresponded to infection. Images were obtained on an Olympus BX-50 light microscope at 10-fold original magnifications (10×). Click here for file

## References

[B1] Dawood FS Jain S Finelli L Shaw MW Lindstrom S Garten RJ Gubareva LV Xu X Bridges CB Uyeki TM Emergence of a novel swine-origin influenza A (H1N1) virus in humans N Engl J Med 2009 360 2605 2615 19423869 10.1056/NEJMoa0903810

[B2] Forgie SE Keenliside J Wilkinson C Webby R Lu P Sorensen O Fonseca K Barman S Rubrum A Stigger E Marrie TJ Marshall F Spady DW Hu J Loeb M Russell ML Babiuk LA Swine outbreak of pandemic influenza A virus on a Canadian research farm supports human-to-swine transmission Clin Infect Dis 2011 52 10 18 10.1093/cid/ciq030 21148514 PMC3106227

[B3] Chen H Wang Y Liu W Zhang J Dong B Fan X de Jong MD Farrar J Riley S Smith GJ Guan Y Serologic survey of pandemic (H1N1) 2009 virus, Guangxi Province, China Emerg Infect Dis 2009 15 1849 1850 10.3201/eid1511.090868 19891883 PMC2857250

[B4] Zhou H Wang C Yang Y Guo X Kang C Chen H Jin M Pandemic (H1N1) 2009 virus in swine herds, People’s Republic of China Emerg Infect Dis 2011 17 1757 1759 10.3201/eid1709.101916 21888815 PMC3322066

[B5] Sreta D Tantawet S Na Ayudhya SN Thontiravong A Wongphatcharachai M Lapkuntod J Bunpapong N Tuanudom R Suradhat S Vimolket L Poovorawan Y Thanawongnuwech R Amonsin A Kitikoon P Pandemic (H1N1) 2009 virus on commercial swine farm, Thailand Emerg Infect Dis 2010 16 1587 1590 10.3201/eid1610.100665 20875285 PMC3298289

[B6] Song MS Lee JH Pascua PN Baek YH Kwon HI Park KJ Choi HW Shin YK Song JY Kim CJ Choi YK Evidence of human-to-swine transmission of the pandemic (H1N1) 2009 influenza virus in South Korea J Clin Microbiol 2010 48 3204 3211 10.1128/JCM.00053-10 20610681 PMC2937722

[B7] Howard WA Essen SC Strugnell BW Russell C Barass L Reid SM Brown IH Reassortant Pandemic (H1N1) 2009 virus in pigs, United Kingdom Emerg Infect Dis 2011 17 1049 1052 10.3201/eid1706.101886 21749767 PMC3358214

[B8] Pereda A Rimondi A Cappuccio J Sanguinetti R Angel M Ye J Sutton T Dibárbora M Olivera V Craig MI Quiroga M Machuca M Ferrero A Perfumo C Perez DR Evidence of reassortment of pandemic H1N1 influenza virus in swine in Argentina: are we facing the expansion of potential epicenters of influenza emergence? Influenza Other Respi Viruses 2011 5 409 412 10.1111/j.1750-2659.2011.00246.x 21668680 PMC3175318

[B9] Weingartl HM Berhane Y Hisanaga T Neufeld J Kehler H Emburry-Hyatt C Hooper-McGreevy K Kasloff S Dalman B Bystrom J Alexandersen S Li Y Pasick J Genetic and pathobiologic characterization of pandemic H1N1 2009 influenza viruses from a naturally infected swine herd J Virol 2010 84 2245 2256 10.1128/JVI.02118-09 20015998 PMC2820904

[B10] Xu L Bao L Zhou J Wang D Deng W Lv Q Ma Y Li F Sun H Zhan L Zhu H Ma C Shu Y Qin C Genomic polymorphism of the pandemic A (H1N1) influenza viruses correlates with viral replication, virulence, and pathogenicity in vitro and in vivo PLoS One 2011 6 e20698 10.1371/journal.pone.0020698 21698272 PMC3115934

[B11] Moreno A Di Trani L Faccini S Vaccari G Nigrelli D Boniotti MB Falcone E Boni A Chiapponi C Sozzi E Cordioli P Novel H1N2 swine influenza reassortant strain in pigs derived from the pandemic H1N1/2009 virus Vet Microbiol 2010 149 472 477 21208754 10.1016/j.vetmic.2010.12.011

[B12] Vijaykrishna D Poon LL Zhu HC Ma SK Li OT Cheung CL Smith GJ Peiris JS Guan Y Reassortment of pandemic H1N1/2009 influenza A virus in swine Science 2010 328 1529 10.1126/science.1189132 20558710 PMC3569847

[B13] Zhu H Zhou B Fan X Lam TT Wang J Chen A Chen X Chen H Webster RG Webby R Peiris JS Smith DK Guan Y Novel reassortment of Eurasian avian-like and pandemic/2009 influenza viruses in swine: infectious potential for humans J Virol 2011 85 10432 10439 10.1128/JVI.05352-11 21849442 PMC3187487

[B14] Belser JA Wadford DA Pappas C Gustin KM Maines TR Pearce MB Zeng H Swayne DE Pantin-Jackwood M Katz JM Tumpey TM Pathogenesis of pandemic influenza A (H1N1) and triple-reassortant swine influenza A (H1) viruses in mice J Virol 2010 84 4194 4203 10.1128/JVI.02742-09 20181710 PMC2863721

[B15] Itoh Y Shinya K Kiso M Watanabe T Sakoda Y Hatta M Muramoto Y Tamura D Sakai-Tagawa Y Noda T Sakabe S Imai M Hatta Y Watanabe S Li C Yamada S Fujii K Murakami S Imai H Kakugawa S Ito M Takano R Iwatsuki-Horimoto K Shimojima M Horimoto T Goto H Takahashi K Makino A Ishigaki H Nakayama M In vitro and in vivo characterization of new swine-origin H1N1 influenza viruses Nature 2009 460 1021 1025 19672242 10.1038/nature08260 PMC2748827

[B16] Maines TR Jayaraman A Belser JA Wadford DA Pappas C Zeng H Gustin KM Pearce MB Viswanathan K Shriver ZH Raman R Cox NJ Sasisekharan R Katz JM Tumpey TM Transmission and pathogenesis of swine-origin 2009 A(H1N1) influenza viruses in ferrets and mice Science 2009 325 484 487 19574347 10.1126/science.1177238 PMC2953552

[B17] Munster VJ de Wit E van den Brand JM Herfst S Schrauwen EJ Bestebroer TM van de Vijver D Boucher CA Koopmans M Rimmelzwaan GF Kuiken T Osterhaus AD Fouchier RA Pathogenesis and transmission of swine-origin 2009 A(H1N1) influenza virus in ferrets Science 2009 325 481 483 19574348 10.1126/science.1177127 PMC4814155

[B18] Steel J Staeheli P Mubareka S García-Sastre A Palese P Lowen AC Transmission of pandemic H1N1 influenza virus and impact of prior exposure to seasonal strains or interferon treatment J Virol 2010 84 21 26 10.1128/JVI.01732-09 19828604 PMC2798408

[B19] Ma W Belisle SE Mosier D Li X Stigger-Rosser E Liu Q Qiao C Elder J Webby R Katze MG Richt JA 2009 pandemic H1N1 influenza virus causes disease and upregulation of genes related to inflammatory and immune responses, cell death, and lipid metabolism in pigs J Virol 2011 85 11626 11637 10.1128/JVI.05705-11 21900171 PMC3209293

[B20] Kalthoff D Grund C Harder TC Lange E Vahlenkamp TW Mettenleiter TC Beer M Limited susceptibility of chickens, turkeys, and mice to pandemic (H1N1) 2009 virus Emerg Infect Dis 2010 16 703 705 10.3201/eid1604.091491 20350393 PMC3321957

[B21] Triana-Baltzer GB Gubareva LV Nicholls JM Pearce MB Mishin VP Belser JA Chen LM Chan RW Chan MC Hedlund M Larson JL Moss RB Katz JM Tumpey TM Fang F Novel pandemic influenza A(H1N1) viruses are potently inhibited by DAS181, a sialidase fusion protein PLoS One 2009 4 e7788 10.1371/journal.pone.0007788 19893747 PMC2770640

[B22] Rowe T Banner D Farooqui A Ng DC Kelvin AA Rubino S Huang SS Fang Y Kelvin DJ In vivo ribavirin activity against severe pandemic H1N1 Influenza A/Mexico/4108/2009 J Gen Virol 2010 91 2898 2906 10.1099/vir.0.024323-0 20797971

[B23] Farooqui A Leon AJ Lei Y Wang P Huang J Tenorio R Dong W Rubino S Lin J Li G Zhao Z Kelvin DJ Heterogeneous virulence of pandemic 2009 influenza H1N1 virus in mice Virol J 2012 9 104 10.1186/1743-422X-9-104 22672588 PMC3444956

[B24] Camp JV Chu YK Chung D-H McAllister RC Adcock RS Gerlach RL Wiemken TL Peyrani P Ramirez JA Summersgill JT Jonsson CB Phenotypic differences in virulence and immune response in closely related clinical isolates of influenza A 2009 H1N1 pandemic viruses in mice PLoS One 2013 8 e56602 10.1371/journal.pone.0056602 23441208 PMC3575477

[B25] Reed LJ Muench A simple method of estimating fifty per cent endpoints Am J Epidemiol 1938 27 493 497

[B26] Li Y Zhou H Wen Z Wu S Huang C Jia G Chen H Jin M Transcription analysis on response of swine lung to H1N1 swine influenza virus BMC Genomics 2011 12 398 10.1186/1471-2164-12-398 21819625 PMC3169531

[B27] Akaike T Ando M Oda T Doi T Ijiri S Araki S Maeda H Dependence on O2-generation by xanthine oxidase of pathogenesis of influenza virus infection in mice J Clin Invest 1990 85 739 745 10.1172/JCI114499 2155924 PMC296490

[B28] Garten RJ Davis CT Russell CA Shu B Lindstrom S Balish A Sessions WM Xu X Skepner E Deyde V Okomo-Adhiambo M Gubareva L Barnes J Smith CB Emery SL Hillman MJ Rivailler P Smagala J de Graaf M Burke DF Fouchier RA Pappas C Alpuche-Aranda CM López-Gatell H Olivera H López I Myers CA Faix D Blair PJ Yu C Antigenic and genetic characteristics of swine-origin 2009 A (H1N1) influenza viruses circulating in humans Science 2009 325 197 201 10.1126/science.1176225 19465683 PMC3250984

[B29] Perrone LA Szretter KJ Katz JM Mizgerd JP Tumpey TM Mice lacking both TNF and IL-1 receptors exhibit reduced lung inflammation and delay in onset of death following infection with a highly virulent H5N1 virus J Infect Dis 2010 202 1161 1170 10.1086/656365 20815704 PMC2941567

[B30] Cheung CY Poon LL Lau AS Luk W Lau YL Shortridge KF Gordon S Guan Y Peiris JS Induction of proinflammatory cytokines in human macrophages by influenza A (H5N1) viruses: a mechanism for the unusual severity of human disease? Lancet 2002 360 1831 1837 10.1016/S0140-6736(02)11772-7 12480361

[B31] de Jong MD Simmons CP Thanh TT Hien VM Smith GJ Chau TN Hoang DM Chau NV Khanh TH Dong VC Qui PT Cam BV Ha do Q Guan Y Peiris JS Chinh NT Hien TT Farrar J Fatal outcome of human influenza A (H5N1) is associated with high viral load and hypercytokinemia Nat Med 2006 12 1203 1207 10.1038/nm1477 16964257 PMC4333202

[B32] Svitek N Rudd PA Obojes K Pillet S von Messling V Severe seasonal influenza in ferrets correlates with reduced interferon and increased IL-6 induction Virology 2008 376 53 59 10.1016/j.virol.2008.02.035 18420248

[B33] Garcia-Sastre A Induction and evasion of type I interferon responses by influenza viruses Virus Res 2011 162 12 18 10.1016/j.virusres.2011.10.017 22027189 PMC3640439

[B34] Moore KW de Waal Malefyt R Coffman RL O’Garra A Interleukin-10 and the interleukin-10 receptor Annu Rev Immunol 2001 19 683 765 10.1146/annurev.immunol.19.1.683 11244051

[B35] Zhao G Fan Q Zhong L Li Y Liu W Liu X Gao S Peng D Liu X Isolation and phylogenetic analysis of pandemic H1N1/09 influenza virus from swine in Jiangsu province of China Res Vet Sci 2012 93 125 132 10.1016/j.rvsc.2011.06.009 21723574

[B36] Chutinimitkul S Herfst S Steel J Lowen AC Ye J van Riel D Schrauwen EJ Bestebroer TM Koel B Burke DF Sutherland-Cash KH Whittleston CS Russell CA Wales DJ Smith DJ Jonges M Meijer A Koopmans M Rimmelzwaan GF Kuiken T Osterhaus AD García-Sastre A Perez DR Fouchier RA Virulence-associated substitution D222G in the hemagglutinin of 2009 pandemic influenza A(H1N1) virus affects receptor binding J Virol 2010 84 11802 11813 10.1128/JVI.01136-10 20844044 PMC2977876

[B37] Bussey KA Bousse TL Desmet EA Kim B Takimoto T PB2 residue 271 plays a key role in enhanced polymerase activity of influenza A viruses in mammalian host cells J Virol 2010 84 4395 4406 10.1128/JVI.02642-09 20181719 PMC2863787

[B38] Mehle A Dugan VG Taubenberger JK Doudna JA Reassortment and mutation of the avian influenza virus polymerase PA subunit overcome species barriers J Virol 2011 86 1750 1757 22090127 10.1128/JVI.06203-11 PMC3264373

[B39] Hale BG Steel J Medina RA Manicassamy B Ye J Hickman D Hai R Schmolke M Lowen AC Perez DR García-Sastre A Inefficient control of host gene expression by the 2009 pandemic H1N1 influenza A virus NS1 protein J Virol 2010 84 6909 6922 10.1128/JVI.00081-10 20444891 PMC2898253

