# Supplementary figures and images for: The 2009 pandemic (H1N1) viruses isolated from pigs show enhanced pathogenicity in mice
Source: Vet Res. 2013 Jun 11;44(1):41. doi: 10.1186/1297-9716-44-41 (PMC3686621; doi:10.1186/1297-9716-44-41)

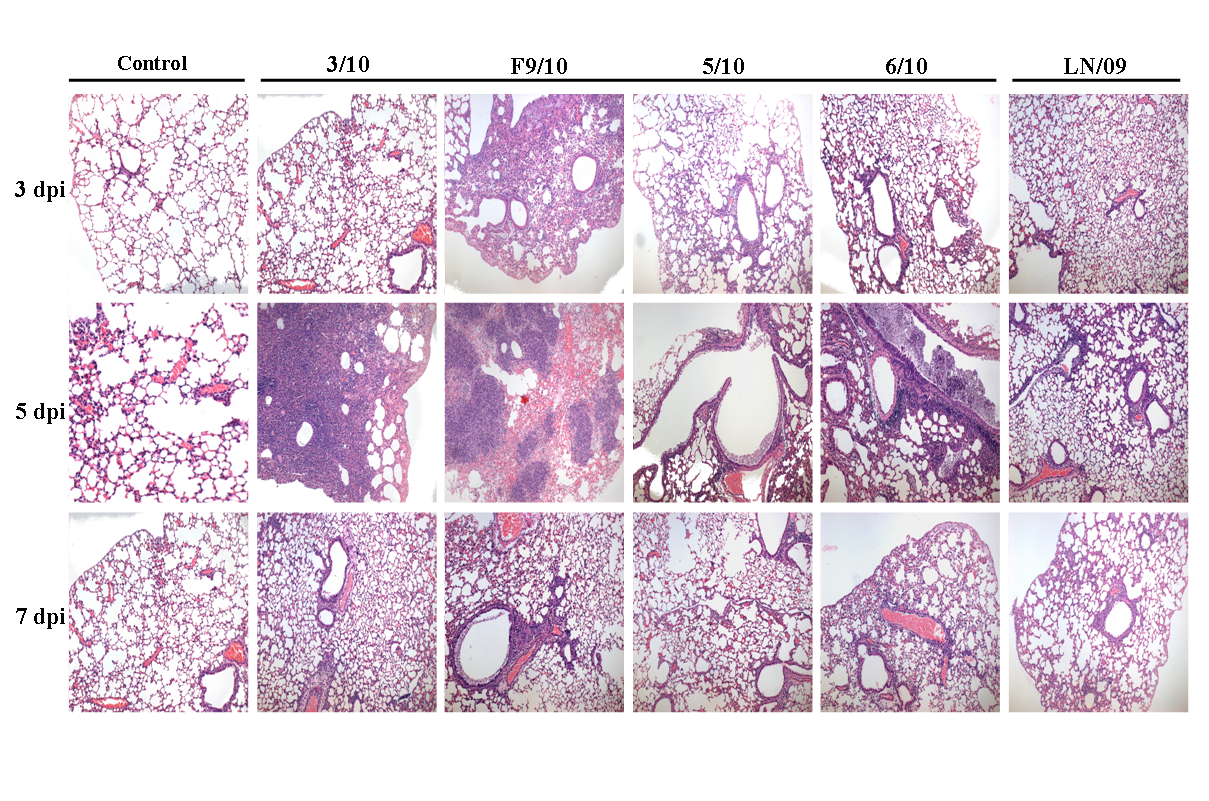

Supplement: Additional file 1 — Lung pathology in BALB/c mice infected with different H1N1/2009 viruses. The formalin-preserved lung samples of mice infected with the four H1N1/2009 pig isolates (3/10, F9/10, 5/10 and 6/10), a human isolate designed LN/09 and control mice were embedded in paraffin and sectioned on 3, 5 and 7 dpi. Serial 4-mm sections were stained with Hematoxylin and Eosin (H&E), and examined for pathological changes that corresponded to infection. Images were obtained on an Olympus BX-50 light microscope at 10-fold original magnifications (10×). [file 1297-9716-44-41-S1.tiff]
